# The Relationship Between Social Vulnerability and COVID-19 Incidence Among Louisiana Census Tracts

**DOI:** 10.3389/fpubh.2020.617976

**Published:** 2021-01-20

**Authors:** Erin N. Biggs, Patrick M. Maloney, Ariane L. Rung, Edward S. Peters, William T. Robinson

**Affiliations:** ^1^Epidemiology Program, Louisiana State University Health Sciences Center School of Public Health, New Orleans, LA, United States; ^2^Division of Global HIV/TB, Centers for Disease Control and Prevention, Epidemiology and Surveillance Branch, Atlanta, GA, United States; ^3^Behavioral and Community Health Sciences, Louisiana State University Health Sciences Center School of Public Health, New Orleans, LA, United States; ^4^STD/HIV Program, Louisiana Office of Public Health, New Orleans, LA, United Sates

**Keywords:** social vulnerability, COVID-19, CDC SVI, Louisiana, SARS- CoV2, racial disparities

## Abstract

**Objective:** To examine the association between the Centers for Disease Control and Prevention (CDC)'s Social Vulnerability Index (SVI) and COVID-19 incidence among Louisiana census tracts.

**Methods:** An ecological study comparing the CDC SVI and census tract-level COVID-19 case counts was conducted. Choropleth maps were used to identify census tracts with high levels of both social vulnerability and COVID-19 incidence. Negative binomial regression with random intercepts was used to compare the relationship between overall CDC SVI percentile and its four sub-themes and COVID-19 incidence, adjusting for population density.

**Results:** In a crude stratified analysis, all four CDC SVI sub-themes were significantly associated with COVID-19 incidence. Census tracts with higher levels of social vulnerability were associated with higher COVID-19 incidence after adjusting for population density (adjusted RR: 1.52, 95% CI: 1.41-1.65).

**Conclusions:** The results of this study indicate that increased social vulnerability is linked with COVID-19 incidence. Additional resources should be allocated to areas of increased social disadvantage to reduce the incidence of COVID-19 in vulnerable populations.

## Introduction

On March 9, 2020, the first presumptive COVID-19 case was reported in Louisiana (1). Throughout the Summer of 2020, Louisiana has remained an epicenter for COVID-19 in the United States with 2,495 reported cases per 100,000 persons and 86 COVID-19 related deaths per 100,000 persons, which are currently some of the highest incidence and mortality rates in the United States (2). As of September 3, 2020, there has been a cumulative total of 150,651 reported COVID-19 cases and 4,858 COVID-19 related deaths in Louisiana (3). In addition, 38% of reported COVID-19 cases and 47% of COVID-19-related deaths are among Black people, yet Black people make up just 33% of the Louisiana population. Similar racial disparities in COVID-19 outcomes have been found across the United States (US), where Black people are over 2.6 times more likely to be an incident COVID-19 case and 2.1 times more likely to die from COVID-19 than non-Hispanic Whites (4). Neighborhood level factors, such as social vulnerability, could explain why Black people and other minorities may be more impacted from the COVID-19 pandemic than other races. Social vulnerability is defined as the degree to which a community is able to prepare and respond to a natural or man-made disaster, such as a hurricane, chemical spill, or disease outbreak (5). Secondary to a history of racial discrimination and residential segregation, Black people and other minorities tend to reside in neighborhoods of higher social vulnerability, which may have contributed to their limited capacity to prepare and respond to the COVID-19 pandemic (6, 7). Race, ethnicity, income, education, household composition, and transportation are factors that influence neighborhood social vulnerability (8). Recent literature suggests that Black people and other minorities are more likely to have limited financial resources and insecure housing, which may impact their ability to properly social distance and self-isolate (7, 9, 10). Essential workers and public transit riders are disproportionally composed of racial minorities, which may increase their risk of exposure to and subsequent infection with COVID-19 (7, 10, 11).

Kim and Bostwick (6) reported a positive correlation between the proportion of Black people in Chicago census tracks and social vulnerability, and they also observed that there were spatial clusters of social vulnerability, which were associated with increased COVID-19 related death rates. Other studies have also found that a positive relationship between social vulnerability and COVID-19 mortality among US census tracts (12, 13). However, to date, there have been mixed results on the relationship between social vulnerability and cumulative incidence of confirmed COVID-19 cases (12, 13). Among US counties, one study observed an association between overall county-level social vulnerability and COVID-19 cumulative incidence between January 21, 2020, and May 12, 2020 but this relationship varied among counties based on a geographically weighted model (12). On the contrary, another study among 433 US counties did not find an association between county-level social vulnerability and COVID-19 cumulative incidence as of April 4th, 2020 (13). However, social vulnerability may not be homogeneous across the entire geographic area of a county, and census tract level social vulnerability may provide better inferences on the relationship between neighborhood level social vulnerability and COVID-19 incidence.

There has been limited research on the relationship between social vulnerability and COVID-19 incidence at the census tract level. Using CDC's Social Vulnerability Index (SVI), this study will examine the relationship between census tract level social vulnerability and the cumulative incidence of reported COVID-19 cases as of August 23, 2020 among Louisiana census tracts. We hypothesize that census tracts with higher levels of social vulnerability will experience higher rates of incident reported COVID-19 cases. In addition, we will examine each of the four CDC SVI four subthemes (socioeconomic status, household composition and disability, minority status and language, and housing type and transportation) as independent predictors of reported COVID-19 incidence among Louisiana census tracts. With limited treatment options for COVID-19, the public health strategy has been to reduce the transmission of COVID-19 through nonpharmaceutical interventions, such as social distancing, mask mandates, school closures, and limiting gatherings. The impact of our results may help identify neighborhoods more susceptible to COVID-19 infection; these areas may require additional planning and resources to mitigate the current COVID-19 pandemic.

## Methods

### COVID-19 Cumulative Incidence

The primary outcome in this study is COVID-19 cumulative incidence from March 9, 2020 to August 24, 2020 for Louisiana census tracts. The number of COVID-19 cases in each Louisiana census tract is publicly available through the Louisiana Department of Health (LDH) and updated on a weekly basis (3). A COVID-19 case is defined as having a positive COVID-19 confirmatory test result for the detection of SARS-CoV-2 RNA in a clinical specimen using a molecular amplification detection either at the LDH Office of Public Health Laboratory or through commercial labs (3). Confirmed cases containing complete address information were matched to census tracts and included for analysis (82.6%). To preserve confidentiality, LDH excludes census tracts with fewer than 1,000 residents and presents census tracts with five or fewer cases as a range of cases (1 to 5 cases). For the purposes of this analysis, census tracts with five or fewer cases (*n* = 3 census tracts) were considered to have 3 total cases. For 1,105 census tracts, COVID-19 cumulative incidence (cases per 1,000 persons) was calculated by dividing the total number of confirmed COVID-19 cases in the census tract by the total population of the census tract in 2018 and multiplying by 1,000. The population estimate for each census tract is provided in the CDC SVI 2018 database and derived from the U.S. Census Bureau's American Community Survey's (ACS) 5-year (2013-2018) estimates. At the time of this analysis, these were the most recently published ACS 5-year population estimates for Louisiana census tracts. According to the US Census Bureau, the ACS 5-year estimates are best used when examining census tracts because 1-year estimates are not available at this level (14). The overall Louisiana population has remained relatively stable, where between 2018 to 2019 Louisiana lost only 0.2% of its population (or 10,896 persons) (15).

### Social Vulnerability Index

The Centers for Disease Control and Prevention (CDC)'s Social Vulnerability Index (SVI) is a publicly available online tool developed by the Agency for Toxic Substance and Disease Registry's Geospatial Research, Analysis and Services Program (5). The purpose of the CDC SVI is to help identify and map communities that will require support in preparing and responding to disasters. For this analysis, the latest available CDC SVI 2018 database was used. The CDC SVI is constructed using 15 census tract level variables (Table 1) from the American Community Survey 2014-2018 (5-year) data. For each census tract, raw data and percentage for each variable are available. Each of the 15 variables was ranked from highest to lowest vulnerability across all census tracts in Louisiana with a nonzero population, and a percentile rank for each variable was calculated for every census tract. Related variables were grouped into four themes: [1] socioeconomic status, [2] household composition and disability, [3] minority status and language, and [4] housing type and transportation. Table 1 shows the variables that comprise each of the four themes. Percentile rankings for each theme were calculated by summing the percentiles for the variables within in each theme. To construct an overall SVI ranking, the values for all four themes are summed for each Louisiana census tract and then ordered. Percentile ranking values range from 0 to 1, where higher values indicate greater vulnerability. More information about the methods related to the CDC SVI can be found elsewhere (5).

**Table 1:** Descriptive statistics for Louisiana census tracts (*N* = 1,105 census tracts^a^).

	**Variable**	**Mean (SD)**	**Range**
	Cumulative COVID-19 Cases per 1,000 persons^b^	26.1 (11.3)	0-121.7
	Total Population	4,205 (2,301)	1,014-18,524
	Population Density (Residents/Sq.-Mi)	2,559 (3,136)	3-18,400
	Percentage Living Below Poverty Line	21.4% (12.8)	0.0-72.0%
	Unemployment Rate	7.6% (4.9)	0.0-27.2%
Theme 1: Socioeconomic	Per Capita Income	$26,643 (12,280)	$6,651-$97,982
	Percentage with No High School Diploma	16.0% (8.8)	0.0-46.6%
	Percentage of persons aged 65 and older	15.1% (5.2)	0.0-41.7%
	Percentage of persons aged 17 and younger	22.9% (6.2)	0.8-45.3%
Theme 2: Household Composition and Disability	Percentage with a disability	15.5% (5.2)	0.0-36.0%
	Percentage of single parent households	11.1% (6.4)	0.0-41.2%
Theme 3: Minority and Language	Percentage Minority (all persons except non-Hispanic whites)	45.1% (28.9)	0.8%-100%
	Percentage of persons who Speak English “Less than Well”	1.5% (2.6)	0.0-23.2%
	Percentage of housing in structures with 10 or more units	7.0% (12.6)	0.0-91.0%
	Percentage of mobile homes	11.8% (13.6%)	0.0-81.9%
Theme 4: Housing and Transportation	Percentage of occupied housing units with more people than rooms	2.4% (2.3)	0.0-15.0%
	Percentage of households with no vehicle available	10.1% (9.7)	0.0-64.2%
	Percentage of persons in institutionalized group quarters	2.9% (8.7)	0.0-15.0%

a *43 census tracts were excluded due to having a population <1,000 residents or having missing information for the CDC's Social Vulnerability Index*.

b *COVID-19 cumulative incidence was calculated between March 9 to August 24, 2020*.

### Other Covariates

Individual characteristics of cases, such as age, gender, and race were not available in the LDH dataset; therefore, these were not adjusted for this analysis. We were able to adjust for population density of each census tract. Population density may be a confounder in the relationship between social vulnerability and COVID-19 incidence. While overcrowding and sanitation issues are expected to increase the spread of COVID-19 (16), the exact effect of population density on the transmission of COVID-19 remains unclear. However, previous literature had reported areas with higher population densities have been found to have earlier COVID-19 outbreaks than those with lower population densities (17). In addition, areas with a higher population density may have more multi-unit structures and overcrowding, which are measured in the CDC SVI. Thus, population density for each census tract was calculated by dividing its total population in 2018 by its area in square-miles and then log transformed to approximate a normal distribution.

### Statistical Analysis

**Figure 1:**
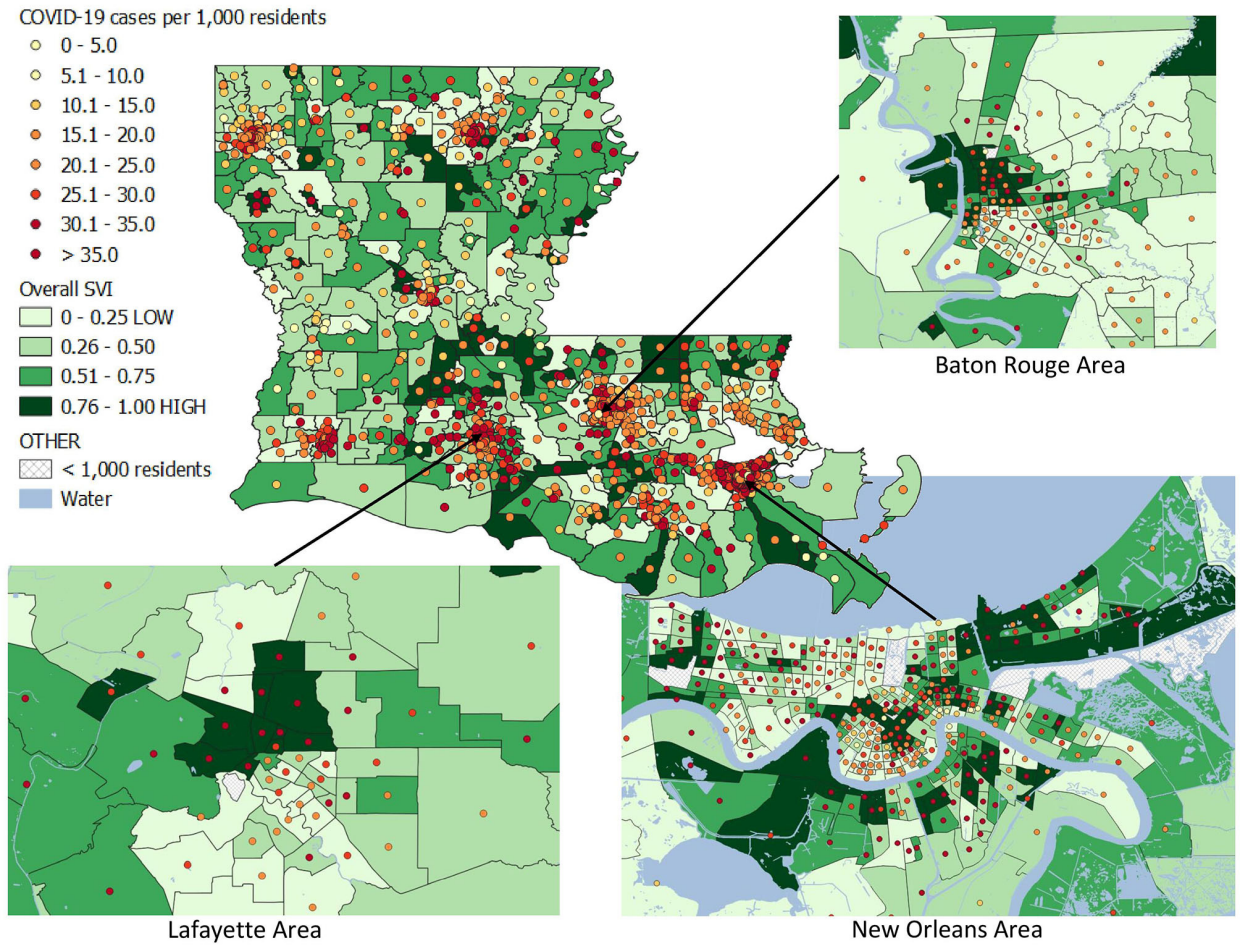
Bivariate map of COVID-19 incidence as of August 24, 2020 and CDC Social Vulnerability Index (2018) percentiles among Louisiana census tracts.

Descriptive statistics, including the mean, standard deviation, and range for the 15 variables used in the CDC SVI, COVID-19 incidence, total population, and population density were calculated. A bivariate map displaying both COVID-19 cumulative incidence and overall SVI ranking was created (Figure 1). Since census tracts in urban areas have smaller geographical areas, subset bivariate maps were constructed for the major cities of New Orleans, Baton Rouge, Lafayette, and their surrounding areas.

For this analysis, negative binomial regression models with random intercepts were performed. The use of random intercept models allows for unspecified noise in our models to account for possible spatial autocorrelation. For all regression models, the natural log of the total population divided by 1,000 for each census tract was used as an offset variable to predict reported COVID-19 cases per 1,000 persons. Crude relative risks (cRR) and 95% confidence intervals (CI) were calculated to measure the associations for all four CDC SVI subtheme rankings and overall SVI ranking with COVID-19 cumulative incidence. A multivariable regression (Model 1) was performed to calculate the adjusted relative risk (aRR) and 95% confidence intervals for the association between overall SVI ranking and COVID-19 incidence after adjusting for population density. A second multivariable regression (Model 2) included all four SVI sub-theme rankings and population density.

The bivariate map was created using QGIS 3.12.2 and the remaining statistical analysis was performed in SAS software 9.4.

## Results

The final analytic sample size was 1,105 census tracts (96.3% of all Louisiana census tracts). A total of 43 tracts were excluded, where 42 tracts had a population <1,000 residents and one additional tract was missing information on the CDC SVI. The descriptive statistics for the study sample are displayed in Table 1. The mean cumulative incidence of confirmed COVID-19 cases per census tract was 26.1 cases per 1,000 residents (SD: 11.3). The mean percentage of the population living below the poverty line is 21.4% (SD: 12.8%) and the average per capita income is $26,643 (SD: $12,280) dollars per year for Louisiana census tracts. In addition, the mean percentage of the population ages 65 and older is 15.1% (SD: 5.2%) and the mean percentage of the population that identifies as a minority race is 45.1% (SD: 28.9%) in Louisiana census tracts.

Figure 1 is a bivariate map for overall SVI rankings and COVID-19 cumulative incidence among Louisiana census tract. The color of the census tract represents the overall SVI ranking for that census tract, where darker green categories represents areas with the higher levels of social vulnerability. The color of the dot represents the COVID-19 incidence for that tract, where the light-yellow color represents areas with the lowest COVID-19 incidence and dark red colors present the highest COVID-19 incidence. Figure 1 also presents magnified bivariate maps for Baton Rouge, New Orleans, and Lafayette and their surrounding areas, since these areas have tracts with small geographic areas. In these maps, many census tracts with the highest level of social vulnerability (0.76–1.00) are also those census tracts with the highest COVID-19 incidence (> 35.0 cases per 1,000 residents).

The regression results are presented in Table 2. Based on the crude model, the overall SVI ranking was significantly associated with COVID-19 incidence; for every percentile increase in overall SVI ranking, COVID-19 cumulative incidence increases by multiplicative factor of 1.52 (cRR: 1.52, 95% CI: 1.40-1.65) before adjusting for population density. All four social vulnerability sub-theme rankings were found to have a positive association with COVID-19 incidence prior to adjusting for any other covariates. Subtheme 3, measuring minority and language, had the strongest relationship with COVID-19 incidence (cRR: 1.60, 95% CI: 1.48-1.74). The other three themes, measuring socioeconomic status (subtheme 1), house composition & disability (subtheme 2), and housing & transportation (subtheme 4) were also associated with an increase in COVID-19 incidence prior to adjusting for other covariates (*p* < 0.01). Greater population density as well was found to have a positive association with COVID-19 incidence (cRR: 1.06, 95% CI: 1.04-1.07).

**Table 2:** Relative risks of COVID-19 cumulative incidence from March 9 to August 24, 2020 among Louisiana Census Tracts (*N* = 1,105 census tracts).

**Variable**	**Crude RR (95% CI)**	**Model 1^**a**^ adjusted RR (95% CI)**	**Model 2^**b**^ adjusted RR (95% CI)**
Overall Social Vulnerability Index	1.52 (1.40-1.65)	1.52 (1.41-1.65)	-
Theme 1 - Socioeconomic	1.32 (1.21-1.44)	-	0.97 (0.87-1.09)
Theme 2-House Composition & Disability	1.27 (1.17-1.39)	-	1.24 (1.12-1.36)
Theme 3- Minority & Language	1.60 (1.48-1.70)	-	1.36 (1.24-1.49)
Theme 4- Housing & Transportation	1.35 (1.24-1.46)	-	1.21 (1.10-1.32)
Log(Population Density)	1.06 (1.04-1.07)	1.06 (1.04-1.07)	1.05 (1.03-1.06)

*RR, Relative Risk; CI, Confidence Interval*.

a *Model 1 includes the CDC's Overall Social Vulnerability Index and natural log of the population density for each census tract*.

b *Model 2 includes all four CDC Social Vulnerability Index subthemes and the natural log of the population density for each census tract*.

*Note: Relative risks and 95% confidence intervals were estimated using negative binomial regression with random intercepts. For all regression models, the natural log of the total population divided by 1,000 for each census tract was used as an offset variable to predict reported COVID-19 cases per 1,000 persons*.

After adjusting for population density, the positive association between overall SVI ranking and COVID-19 incidence remained (Model 1), suggesting that population density does not explain the relationship between SVI and COVID-19. For each percentile increase in overall SVI, the COVID-19 incidence increased by a multiplicative factor of 1.52 (95% CI: 1.41-1.65) after adjusting for population density. While population density was found to be significant predictor of COVID-19 incidence in Model 1, the relative risk for the association between overall SVI and COVID-19 remained the same. Therefore, population density was not found to distort this relationship.

When including all four CDC SVI subthemes and population density in Model 2, three of the four subthemes were observed to have a positive association with COVID-19 cumulative incidence. Subtheme 3, measuring minority status and language was found to have the strongest relationships with COVID-19 incidence (aRR: 1.36, 95% CI: 1.12-1.49) after adjusting for the other SVI subthemes and population density. In addition, subtheme 2, measuring house composition and disability and subtheme 4, measuring housing and transportation remained positively associated with COVID-19 incidence after adjusting for the other SVI subthemes and population density (aRR: 1.24, 95% CI: 1.12-1.26; aRR: 1.21, 95% CI: 1.10-1.32, respectively). However, the relationship between SVI subtheme 1, measuring socioeconomic status, and COVID-19 incidence was no longer observed after adjusting for the other three SVI subthemes and population density (aRR: 0.97, 95% CI: 0.87-1.09).

## Discussion

In summary, our results showed that overall social vulnerability was positively associated with reported COVID-19 cumulative incidence at the census tract level even after controlling for population density. In addition, we found that all four CDC SVI subthemes were positively associated with COVID-19 cumulative incidence before adjusting for other covariates. After adjusting for all four CDC SVI subthemes and population density, theme 2, measuring house composition and disability, theme 3, measuring minority and language, and theme 4, measuring housing and transportation, were found to have the positive association with COVID-19 cumulative incidence.

The results of our study are reflective of the long history of racial residential segregation in the United States, where Black people and other minorities are concentrated in neighborhoods of high levels of social vulnerability (18). Residence in these disadvantaged neighborhoods leads to attendance at poorer quality schools, which leads to lower educational attainment, which influences household income, which determines the neighborhoods in which families are able to reside as well as their housing characteristics (e.g., multiunit rental properties, overcrowding, etc.) (7). This enduring cycle of poverty is especially prevalent among racial minority populations, who have disproportionately higher rates of poverty, lower household income, and lower educational attainment than Whites (7, 9). While theme 3, measuring minority status and language, was one of the strongest predictors of COVID-19 incidence, all four of the CDC SVI subthemes are interconnected, and the compound effects of these factors may impact the likelihood of COVID-19 infection.

The combination of these interlinking factors contributes to increased COVID-19 incidence in areas with high social vulnerability. Persons with lower income and no high school diploma are more likely to be an hourly-paid essential employee; thus, they are not physically or financially able to work remotely from home, where they would be more protected from contracting the disease (7, 9, 10). In addition, the COVID-19 pandemic has been regarded as an “infodemic” due to the propagation of misinformation on social media, which may have a greater impact on low socioeconomic individuals. Previous researchers have reported that quality scientific information is more likely to reach more educated and high-income persons, and that these groups also process new information more efficiently (19). Furthermore, persons living below the poverty line may have unstable housing and lack access to clean water, which will limit proper hygiene (7, 20). Low socioeconomic individuals may have limited financial resources to purchase disposable face masks, and they may not have the ability to wash reusable cloth masks as often as needed. They may also rely on public laundromats, which increases their contact rate with others.

In addition, household composition is a key factor in transmission of COVID-19. For example, crowded homes with large families who share rooms may struggle to practice proper social distancing and self-isolation, which will increase the risk of in-home transmission of COVID-19 (9, 10). In particular, multigenerational homes may increase the risk of contracting COVID-19 among elderly persons living in the home, who are at higher risk for severe COVID-19 infection. In fact, Black people and Hispanics are more likely to live in a multigenerational home than Whites (21). In addition, those living in multi-unit structures may have shared facilities (such as laundry room, mail rooms), which could increase a person's contact rate with others. Finally, those with without a vehicle or those with a disability who are unable to drive may rely on public transit, which also increases their risk of contracting COVID-19 (7, 10, 11). The combination of these four factors: [1] socioeconomic status, [2] household composition & disability, [3] minority & language, and [4] housing type and transportation leads to an increase in COVID-19 transmission, which in turn leads to higher COVID-19 prevalence in the community; thus, there is an increased likelihood of COVID-19 exposure among those still at risk for COVID-19.

Previous studies examining the association between CDC SVI and COVID-19 cumulative incidence have had mixed results at the county level. As mentioned earlier, Karaye and Horney reported that there was positive relationship between overall county-level social vulnerability and COVID-19 cumulative incidence that varied spatially among US counties (12). In addition, Nayak et al. (13) did not observe an association between overall CDC SVI and COVID-19 cumulative incidence among US counties (aRR: 1.30; 95% CI: 0.95-1.78). Both studies found that CDC SVI subtheme 3, measuring minority status and language, was associated with greater COVID-19 infections, which aligns with our study results. However, both studies measured CDC SVI and COVID-19 incidence at the county level, while our study measured these at the census tract level. On the census tract level, Kim and Bostwick (6) found a positive association between social vulnerability and COVID-19 incidence among Chicago census tracts; however, they did not use the CDC SVI to measure social vulnerability.

Our study had a large sample of 1,105 census tracts within Louisiana with a racially diverse population compared to other areas in the United States. Furthermore, our study utilized the CDC's Social Vulnerability Index database, which includes social vulnerability rankings for all US census tracts; thus, this study can be readily repeated at a multistate or national level. Due to the cross-sectional nature of the case data, our study measured COVID-19 cumulative incidence at a single time-point; thus, we were not able to explore time-varying effects in the association between social vulnerability and COVID-19 incidence, which may occur secondary to the implementation and relaxation of nonpharmaceutical interventions. In addition, we were also not able to adjust for COVID-19 testing practices within census tracts. Finally, our study does not account for underlying health conditions (i.e., chronic kidney disease, cancer, coronary artery disease and etc.) that increase COVID-19 severity and mortality (22), in which these factors may vary by census tracts. Persons with a more severe COVID-19 case may be more likely to seek testing and thus be diagnosed with COVID-19. However, previous literature has found that social vulnerability is positively associated with the overall prevalence of comorbidities (6). Therefore, underlying health conditions may be an intermediate step in the casual pathway between social vulnerability and COVID-19 morbidity and mortality.

## Conclusion

These results offer preliminary findings at the ecological level, suggesting future multilevel studies to examine the effects of social vulnerability on COVID-19 outcomes while accounting for individual characteristics. Furthermore, future studies should also examine the potential impact of non-pharmaceutical interventions on the relationship between social vulnerability and COVID-19 incidence. Throughout, the course of the epidemic, several nonpharmaceutical interventions have been implemented, such as stay-at-home orders, school closures, and mask mandates. Therefore, a longitudinal analysis may allow for researchers to examine how social vulnerability impacted COVID-19 incidence following the introduction and relaxation of these interventions.

In conclusion, we found a positive association between the CDC Social Vulnerability Index and cumulative COVID-19 incidence, in which areas with higher social vulnerability were found to have higher COVID-19 incidences. The CDC's Social Vulnerability Index could be useful in identifying locations that are most impacted by COVID-19 and should thus be targeted for more specific interventions. The factors that comprise social vulnerability, such as income, education, poverty, race, and ethnicity influence who will suffer the most from the COVID-19 epidemic (23). The findings in this paper support the recent argument presented by Rollston and Galea (7) that the United States faces significant challenges in its handling of the COVID-19 epidemic, particularly due to the nation's structural racism and inattention to the barriers to health which are at the root of racial health disparities across the nation (18). Policy initiatives are needed to provide additional resources and planning to not only reduce and cease COVID-19 transmission but to also address the financial and emotional distress following the COVID-19 epidemic among the most socially vulnerable populations.

## Data Availability Statement

Publicly available datasets were analyzed in this study. This data can be found here: The Louisiana Department of Public Health. COVID-19. https://ladhh.maps.arcgis.com/apps/webappviewer/index.html?id=3b9b6f22d92f4d688f1c21e9d154cae22. The Centers for Disease Control and Prevention. Agency for Toxic Substances and Disease Registry. CDC's Social Vulnerability Index (SVI). https://www.atsdr.cdc.gov/placeandhealth/svi/data_documentation_download.html.

## Ethics Statement

Ethical review and approval was not required for the study on human participants in accordance with the local legislation and institutional requirements. Written informed consent for participation was not required for this study in accordance with the national legislation and the institutional requirements.

## Author Contributions

All authors contributed significantly to the project and writing of the manuscript. All authors reviewed the final paper and provided comments as deemed necessary. EB conducted the formal analysis and wrote the original draft. PM assisted with the methodology and provided review and editing. AR and EP provided crucial feedback and revisions to the original draft. WR conceptualized the original idea, assisted with the methodology, and supervised the project.

## Conflict of Interest

The authors declare that the research was conducted in the absence of any commercial or financial relationships that could be construed as a potential conflict of interest. The reviewer LC declared a shared affiliation with one of the authors, PM at time of review.

## References

[1] Government LS Gov. Edwards Confirms Louisiana's First Presumptive Positive Case of COVID-19. (2020). Available online at: https://gov.louisiana.gov/index.cfm/newsroom/detail/2392

[2] Centers for Disease for Control and Prevention CDC COVID-19 Data Tracker. (2020). Available online at: https://www.cdc.gov/covid-data-tracker/#cases

[3] Louisiana Department of Health COVID-19. (2020). Available online at: https://ldh.la.gov/coronavirus/

[4] Division of Viral Diseases, National Center for Immunization and Respiratory Diseases (NCIRD) Centers for Disease Control and Prevention COVID-19 Cases, Hospitalization, and Death By Race/Ethnicity. Atlanta, GA (2020). Available online at: https://www.cdc.gov/coronavirus/2019-ncov/covid-data/investigations-discovery/hospitalization-death-by-race-ethnicity.html

[5] Centers for Disease Control and Prevention (CDC) Geospatial Research A& SP (GRASP). CDC's Social Vulnerability Index (SVI) 2016 Documentation. (2018). p. 1–24. Available online at: https://svi.cdc.gov/data-and-tools-download.html

[6] Kim SJ Bostwick W. Social vulnerability and racial inequality in COVID-19 deaths in Chicago. Heal Educ Behav. (2020) 47:509–13. 10.1177/1090198120929677 32436405 PMC8183499

[7] Rollston R Galea S. COVID-19 and the social determinants of health. Am J Heal Promot. (2020) 34:687–9. 10.1177/0890117120930536b 32551932

[8] Flanagan BE Hallisey EJ Adams E Lavery A. Measuring community vulnerability to natural and anthropogenic hazards: the centers for disease control and prevention's social vulnerability index. J Environ Health. (2018) 80:34–6. 32327766 PMC7179070

[9] van DR Cooney RE Sabin ML. COVID-19 exacerbating inequalities in the US. Lancet. (2020) 395:1243–4. 32305087 10.1016/S0140-6736(20)30893-X PMC7162639

[10] Smith JA Judd J. COVID-19: vulnerability and the power of privilege in a pandemic. Health Promot J Austr. (2020) 31:158–60. 10.1002/hpja.333 32197274 PMC7165578

[11] Sy KTL Martinez ME Rader B Laura F White LF Socioeconomic disparities in subway use and COVID-19 outcomes in New York city. Am J Epidemiol. (2020). 10.1093/aje/kwaa277. [Epub ahead of print]. PMC7799254 33372209

[12] Karaye IM Horney JA. The impact of social vulnerability on COVID-19 in the U.S.: An analysis of spatially varying relationships. Am J Prev Med. (2020) 59:317–25. 10.1016/j.amepre.2020.06.006 32703701 PMC7318979

[13] Nayak A Islam SJ Mehta A Ko Y-A Patel SA Goyal A. Impact of social vulnerability on COVID-19 incidence and outcomes in the United States. medRxiv [Preprint]. (2020). 10.1101/2020.04.10.20060962 32511437 PMC7217093

[14] U.S. Census Bureau When to Use 1-year, 3-year, or 5-year Estimates. Available online at: https://www.census.gov/programs-surveys/acs/guidance/estimates.html

[15] U.S. Census Bureau 2019 U.S. Population Estimates Continue to Show the Nation's Growth Is Slowing. Available online at: https://www.census.gov/newsroom/press-releases/2019/popest-nation.html

[16] Garg R Feigin RD Encyclopædia Britannica, Inc. Available online at: https://www.britannica.com/science/infectious-disease

[17] Carozzi F Provenzano S Roth S Urban Density and COVID-19. Bonn: Institute of Labor Economics.

[18] Williams DR Collins C. Racial residential segregation: a fundamental cause of racial disparities in health. Public Health Rep. (2001) 116:404–16. 10.1093/phr/116.5.404 12042604 PMC1497358

[19] Scheufele DA Krause NM. Science audiences, misinformation, and fake news. Proc Natl Acad Sci USA. (2019) 116:7662–9. 10.1073/pnas.1805871115 30642953 PMC6475373

[20] Lancet T. Redefining vulnerability in the era of COVID-19. Lancet. (2020) 395:1089. 10.1016/S0140-6736(20)30757-1 32247378 PMC7270489

[21] Kochhar R Cohn D Fighting Poverty in a Bad Economy, Americans Move in with Relatives. (2011). Available online at: http://www.pewsocialtrends.org/2011/10/03/fighting-poverty-in-a-bad-economy-americans-move-in-with-relatives/

[22] Centers for Disease Control and Prevention COVID-19: People with Certain Medical Conditions. (2020). Available online at: https://www.cdc.gov/coronavirus/2019-ncov/need-extra-precautions/people-with-medical-conditions.html#:~:text=People of any age with the following conditions are at,COPD (chronic obstructive pulmonary disease)

[23] Morabia A. COVID-19: health as a common good. Am J Public Health. (2020) 110:1111–2. 10.2105/AJPH.2020.305802 32639904 PMC7349450

